# Temporal Muscle Cavernous Hemangioma: A Case Report and Literature Review

**DOI:** 10.7759/cureus.23166

**Published:** 2022-03-15

**Authors:** Ahmed A Alqahtani, Abdulaziz A AlQarni, Munzir M Abbas, Ahmad M Alkhani

**Affiliations:** 1 Department of Neurosurgery, King Abdulaziz Medical City, Riyadh, SAU

**Keywords:** surgical resection, imaging, intramuscular, temporalis muscle, cavernous hemangioma

## Abstract

Hemangiomas are nonmalignant vascular lesions commonly seen in both the skin and mucosa that rarely occur in skeletal muscles. According to the type of vessel, a hemangioma can be differentiated into capillary, cavernous, or mixed types. The following report describes a case of temporalis muscle hemangioma in a 45-year-old female who presented with painful swelling in the left outer orbital wall, which had been growing in size over the past 12 months. CT scan and MRI revealed a well-demarcated lesion in the temporalis muscle measuring 10 mm × 13 mm × 15 mm. Surgical resection of the lesion was performed, allowing a definitive diagnosis of cavernous hemangioma to be made histologically. As radiological images are mainly inadequate for definitively diagnosing these lesions, surgical intervention is usually required. The recurrence rate differs among the three subtypes; thus, clinical follow-up and radiological imaging as needed are recommended.

## Introduction

Hemangiomas are benign vascular lesions that are classified based on the size of the involved vessels into cavernous (large vessel), capillary (small vessel), and mixed hemangiomas [1,2]. Approximately 0.8% of all hemangiomas are considered intramuscular and are frequently found in the trunk and extremities due to the presence of the large muscles in these regions [3]. Head and neck hemangiomas account for approximately 14% of all hemangiomas, most commonly occurring in the masseters, followed by the trapezius muscles, and very rarely in the temporalis muscle [4]. Unsurprisingly, only 33 cases reported in international literature involve intramuscular hemangioma in the temporalis muscle. Intramuscular hemangiomas usually manifest with slow growth, obvious margins, and painless intramuscular masses, while the size and number can vary considerably. Between 15-25% are also characterized by phlebolith formation, a calcified thrombus [5]. The following report describes a case of intramuscular cavernous hemangioma in the temporalis muscle, followed by a review of the pertinent literature.

## Case presentation

**Figure 1:**
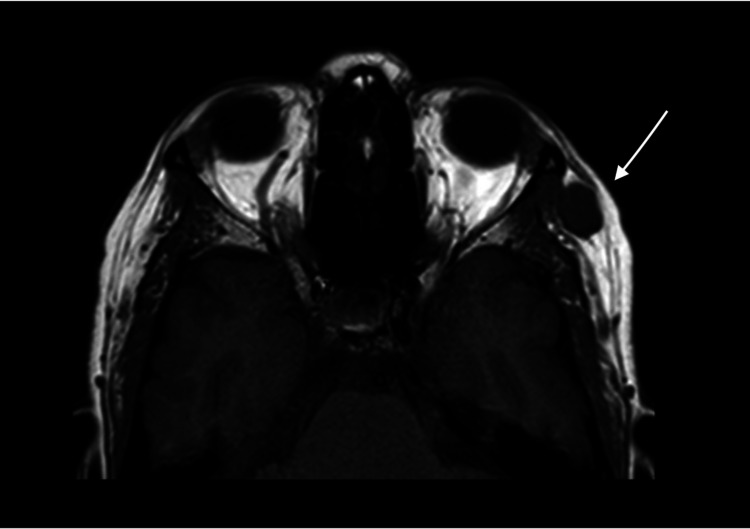
T1-WI imaging, axial view The arrow shows the identified lesion

**Figure 2:**
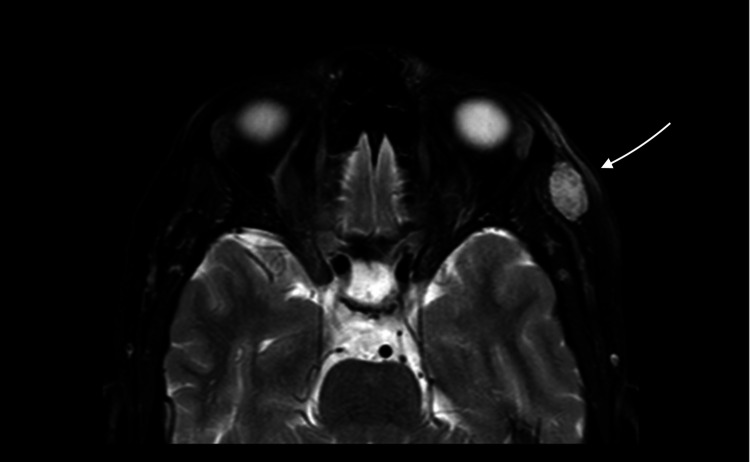
T2-WI imaging, axial view The arrow shows the identified subcutaneous lesion

**Figure 3:**
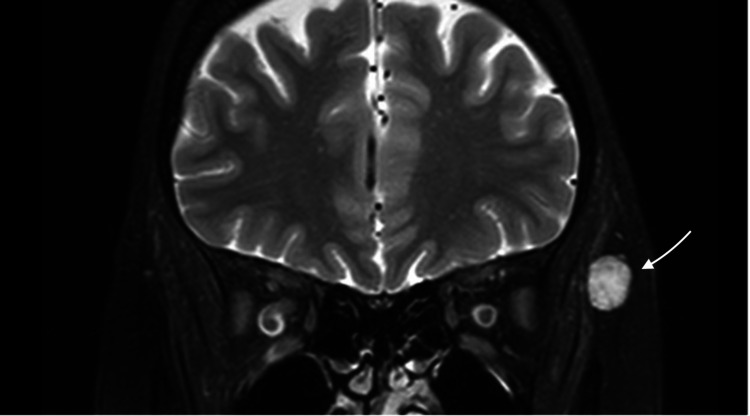
T2-WI imaging, coronal view The arrow shows the identified lesion

A 45-year-old female was admitted to the Neurosurgery Department in King Abdulaziz Medical City, Riyadh, Saudi Arabia. She had a history of painful left orbital swelling on the outer wall that had been progressively increasing in size for a year. She denied any history of trauma, surgery, or visual symptoms. The physical and neurological examinations were unremarkable, with the exception of a smooth and immobile swelling in the outer wall of the left orbit that measured approximately 1.5 cm in diameter and was tender on palpation. No overlying redness or warmth of the skin or any signs or symptoms of inflammation were observed. Additionally, no pulsation or bruit over the lesion was noted. Magnetic resonance imaging (MRI) was performed, revealing a slightly lobulated deep subcutaneous lesion measuring 10 mm × 13 mm × 15 mm in the lateral side of the temporalis muscle at the same level of the left orbit. T1-weighted imaging (T1-WI) exhibited a low signal intensity (Figure 1), while hyperintensity was revealed on T2-WI images (Figures 2, 3).

**Figure 4:**
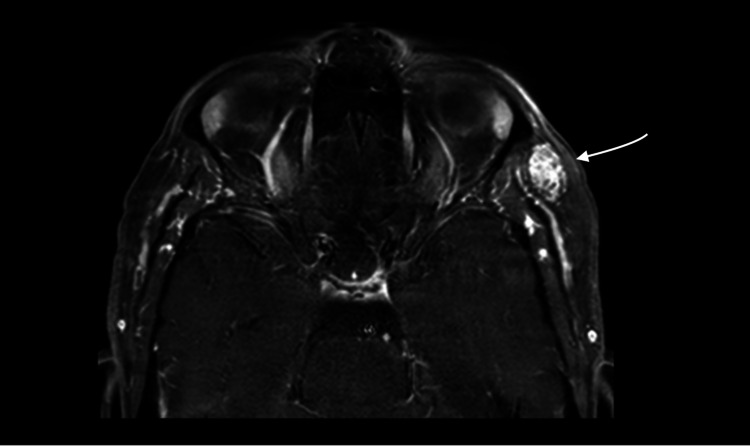
MRI with contrast imaging, axial view The arrow shows the identified lesion

**Figure 5:**
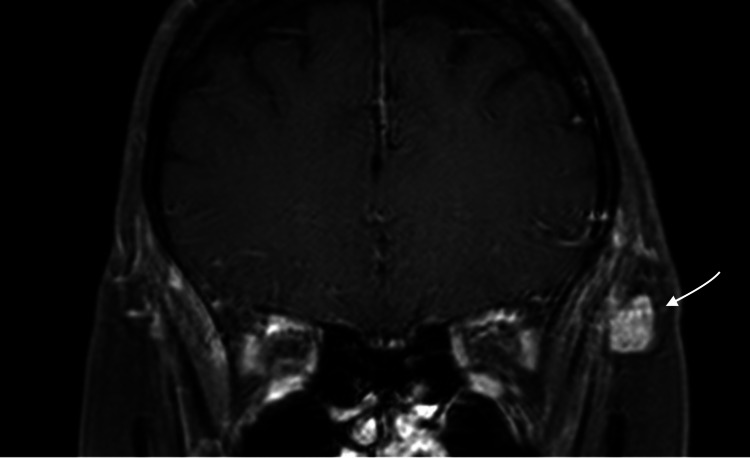
MRI with contrast imaging, coronal view The arrow shows the identified lesion with no calcification

**Figure 6:**
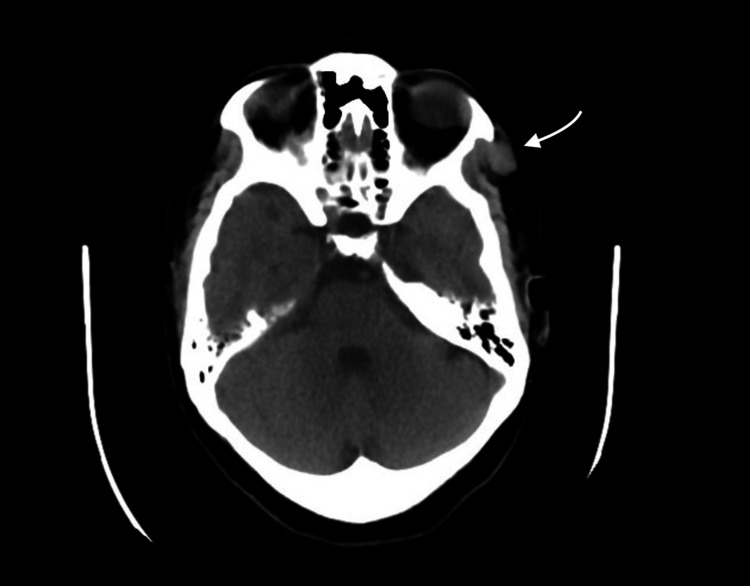
Computed Tomography Scan, axial view The arrow shows the identified lesion

The lesion on contrast-enhanced MRI (Figures 4, 5) appeared with intense and slightly heterogeneous enhancement. A subsequent computed tomography (CT) scan showed the mass to be isodense with no bone erosion and no calcification (Figure 6).

**Figure 7:**
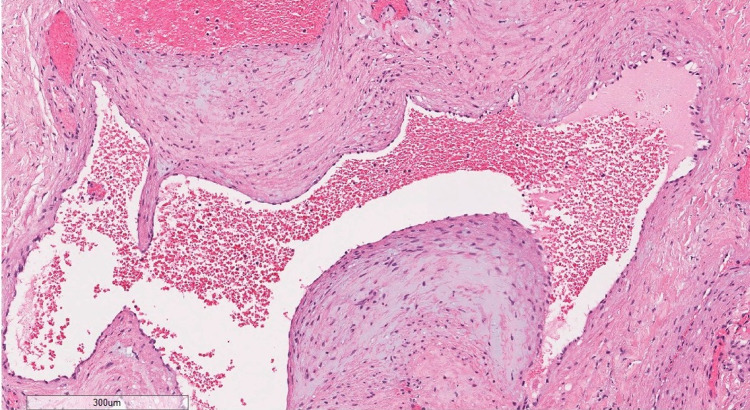
Histopathological Image of the lesion tissue

The patient was taken to the operating room for surgery under general anesthesia. A curvilinear incision was made approximately 1 cm in front of the tragus, and the temporalis muscle and the fascia were dissected by using monopolar electrocautery until a dark-red, soft tissue mass (1.0 cm × 1.0 cm × 0.5 cm) was encountered. Total resection of the lesion with a margin of normal muscle was performed. Histological examination showed a collection of back-to-back cavernous-sized vascular channels. Given the presence of chronic inflammation and epithelioid histiocytes, CD34 and D2-40 immunohistochemistry was performed. The vascular channels were found to be immunopositive for CD34 and immunonegative for D2-40 (Figure 7).

The postoperative events were uncomplicated, and the patient was discharged home in good condition on the second day. Six months of follow-up did not reveal any local recurrence.

## Discussion

Hemangioma is a type of vascular abnormality. Intramuscular hemangioma is a rare benign lesion, as it represents less than 1% of all hemangiomas, 14% of which are found in the head and neck region. The most commonly affected muscles are the masseter (36%) and trapezius (24%) [6], while hemangiomas of the temporalis muscles are extremely rare, as suggested by the limited number (only 33) of cases reported to date in international literature. In 1843, intramuscular hemangioma was primarily reported by Liston [7]. Allen and Enzinger established the most comprehensive classification of intramuscular hemangiomas [1], whereas Enzinger and Weiss subsequently identified three subtypes based on histological findings, denoted as small, large, and mixed-type vascular hemangiomas [8]. Capillary hemangiomas are considered to account for 68% of all intramuscular hemangiomas that involve small capillary-sized vessels, followed by cavernous hemangiomas (with an incidence of 26%), which are distinguished by thin-walled and cystic blood spaces, and finally, venous or mixed-type hemangiomas (6%) that comprise widened veins of irregular thickness inside loose fatty fibrous stroma [9].

The etiology behind these lesions is not known; however, recurrent trauma or hormonal changes can cause the lesion to grow as a result of the proliferation of embryonic vascular tissue [10]. Clinically, in 98% of cases, intramuscular hemangioma presents as a slowly growing mass with definite margins and pain (in 30−60% of the cases) [1,8]. It is mobile and does not demonstrate any vascular signs, such as skin discoloration or pulsation. The most common differential diagnoses include lipoma, lymphoma, dermoid cyst, and temporal arteritis [11,12].

Intramuscular hemangioma is diagnosed preoperatively in only < 8% of cases because of its low incidence and lack of specific symptoms [13,14]; hence, imaging studies play a significant role in the diagnosis. Computed tomography is useful in the determination of lesion size and shape while facilitating the assessment of the surrounding tissues. However, for identifying the nature of the lesion, MRI is mandatory. On T1-WI images, hemangiomas show hypointensity or isointensity with a fat-free lesion, which can exclude angiolipoma. On T2-WI images, the lesion is characterized by hyperintensity and high fluid content. The lesion also demonstrates an enhancement after administration of contrast medium, whereby good vascularization would exclude lymphoma as a potential diagnosis [15]. MRI can also show definitive findings radiologically that favor hemangioma, specifically a hyperintense signal on T2-WI imaging; the presence of fibrosis, thrombus, and/or deposition of hemosiderin; and on histological assessment, endothelial-lined vascular channels are separated by fibrous and fatty linear tissue in considerable lesions measuring 2 cm in diameter [16]. Arteriography may be helpful in identifying the lesion’s feeding vessels and whether these vessels need to be embolized before surgery [17,18].

In the international literature, only 33 cases of temporalis muscle hemangioma have been reported to date (Table 1). All reported cases are characterized by hyperintensity on T2-WI imaging, while T1-WI imaging shows either isointense or hypointense signals. None of the cases involved bony erosion or invasion of the surrounding tissue, but some showed calcification of the lesion on CT. The most common type of hemangioma in the temporalis muscle is cavernous (27/33), followed by capillary (only four cases). The mean age of patients with temporalis muscle hemangioma is 37.76 ± 18.73 years, with a slight male preponderance (18/33). According to the literature review, in 31 of these cases, surgical resection of the lesion was the treatment of choice, while only Heckl et al. and Gadhia et al. followed the patients clinically and radiologically [15,19]. Multiple factors determine if surgical resection is indicated, including the age of the patient, the extent of bleeding, lesion size and location, the level of pain, the depth of invasion, the rate of growth, the degree of cosmetic deformity, and malignant susceptibility [11,13,20]. Local recurrence of the intramuscular hemangioma is associated primarily with incomplete resection, while the rate of local recurrence for completely resected lesions differs among the three pathohistological subtypes, with 28% for capillary, 20% for cavernous, and 9% for mixed-type hemangioma [17]. None of the reported cases of temporalis muscle hemangioma indicated any local recurrence. However, clinical follow-up and radiological images (if needed) are recommended to detect any future recurrence.

**Table 1:** Reported cases of temporalis muscle hemangioma Joehl et al., 1979 [21]; Knox et al., 1990 [6]; Sharma et al., 1991 [22]; Murakami et al., 1991 [23]; Hughes et al., 1993 [24]; Cappabianca et al., 1996 [25]; Lopez-Cedrun et al., 1996 [17]; Tada et al., 1996 [26]; Couloigner et al., 1996 [27]; Shpitzer et al., 1997 [28]; Itosaka et al., 1997 [29]; Benateau et al., 1997 [30]; Sharma et al., 2001 [20]; Sherman & Davies, 2001 [18]; To et al., 2001 [11]; Heckl et al., 2002 [15]; Bui-Mansfield et al., 2002 [12]; Top & Barcin, 2004 [31]; Sakr et al., 2005 [32]; Calişaneller et al., 2007 [33]; Bucci et al., 2008 [34]; Kim, 2009 [35]; Gadhia et al., 2011 [19]; Eryilmaz et al., 2014 [36]; Kim et al., 2014 [37]; Turel et al., 2016 [38]; Cui et al., 2017 [39]; Arora et al., 2017 [40]; Kishimoto et al., 2018 [41]; Jbali et al., 2018 [42]; Motazedian et al., 2019 [43]; Watanabe et al., 2020 [44]

CASE NO.	AUTHOR	GENDER	AGE	SIDE	DIAGNOSED BY	VESSEL TYPE	PLAN
1	Joehl et al., 1979	Female	59 years	Left	Histological	Cavernous	Surgery
2	Knox et al., 1990	Male	19 years	Left	Histological	Cavernous	Surgery
3	Sharma et al., 1991	Male	21 years	Left	Histological	Capillary	Surgery
4	Murakami et al., 1991	Male	51 years	Right	Histological	Cavernous	Surgery
5	Hughes et al., 1993	Female	28 years	Left	Histological	Cavernous	Surgery
6	Cappabianca et al., 1996	Female	13 years	Left	Histological	Cavernous	Surgery
7	Lopez-Cedrun et al., 1996	Male	41 years	Right	Histological	Cavernous	Surgery
8	Tada et al., 1996	Female	14 years	Right	Histological	Cavernous	Surgery
9	Couloigner et al., 1996	Female	41 years	Left	Histological	Cavernous	Surgery
10	Shpitzer et al., 1997	Female	29 years	Right	Histological	Cavernous	Surgery
11	Itosaka et al., 1997	Female	12 years	Right	Histological	Cavernous	Surgery
12	Benateau et al., 1997	Female	61 years	N/A	Histological	Capillary	Surgery
13 – 14	Sharma et al., 2001	Female/Male	5/27 years	Left	Histological	Cavernous/Capillary	Surgery
15	Sherman & Davies, 2001	Male	21 months	Right	Histological	Cavernous	Surgery
16	To et al., 2001	Female	54 years	Right	Histological	Cavernous	Surgery
17	Heckl et al., 2002	Male	55 years	Right	MRI / Clinically	Cavernous	Follow up
18	Bui-Mansfield et al., 2002	Male	44 years	Bilateral	Histological	Cavernous	Surgery
19	Top & Barcin, 2004	Male	46 years	Left	Histological	Mixed Type	Surgery
20	Sakr et al., 2005	Male	44 years	Right	Histological	Cavernous	Surgery
21	Calişaneller et al., 2007	Male	37 years	Right	Histological	Cavernous	Surgery
22	Bucci et al., 2008	Male	38 years	Left	Histological	Cavernous	Surgery
23	Kim, 2009	Male	24 years	Left	Histologically	Cavernous	Surgery
24	Gadhia et al., 2011	Female	57 years	Right	MRI / Clinically	Cavernous	Follow up
25	Eryilmaz et al., 2014	Male	34 years	Left	Histological	Cavernous	Surgery
26	Kim et al., 2014	Female	46 years	Left	Histological	Mixed Type	Surgery
27	Turel et al., 2016	Female	61 years	Left	Histological	Cavernous	Surgery
28	Cui et al., 2017	Male	62 years	Right	Histological	Cavernous	Surgery
29	Arora et al., 2017	Male	5 years	Left	Histological	Capillary	Surgery
30	Kishimoto et al., 2018	Male	43 years	Left	Histological	Cavernous	Surgery
31	Jbali et al., 2018	Female	42 years	Left	Histological	Cavernous	Surgery
32	Motazedian et al., 2019	Male	64 years	Right	Histological	Cavernous	Surgery
33	Watanabe et al., 2020	Female	68 years	Left	Histological	Cavernous	Surgery
34	Our case	Female	45 years	Left	Histological	Cavernous	Surgery

## Conclusions

Hemangiomas are nonmalignant vascular lesions that are uncommonly found in the temporalis muscle. Based on histological findings, they are classified into three subtypes. As radiological images are mainly inadequate for a definitive diagnosis of these lesions, surgical intervention with resection is the treatment of choice in most cases. The recurrence rate of intramuscular hemangioma differs among the three subtypes. Clinical and radiological follow-up and radiological images as needed are recommended.

## References

[1] Allen PW, Enzinger FM (1972). Hemangioma of skeletal muscle. An analysis of 89 cases. Cancer.

[2] Mehrabani D, Tabei SZ, Heydari ST (2008). Cancer occurrence in Fars province, southern Iran. Iran Red Crescent Med J.

[3] Watson WL, Mc Carthy WD (1940). Blood and lymph vessel tumors; a report of 1056 cases. Surg Gynecol Obstet.

[4] Batsakis JG (1979). Tumors of the head and neck: clinical and pathological considerations.

[5] Morris SJ, Adams H (1995). Case report: paediatric intramuscular haemangiomata--don't overlook the phlebolith!. Br J Radiol.

[6] Knox RD, Pratt MF, Garen PD, Giles WC (1990). Intramuscular hemangioma of the infratemporal fossa. Otolaryngol Head Neck Surg.

[7] Liston R (1843). Case of erectile tumour in the popliteal space.-Removal. Med Chir Trans.

[8] Enzinger FM, Weiss SW (1988). Soft tissue tumors. St. Louis: C. V. Mosby.

[9] Beham A, Fletcher CD (1991). Intramuscular angioma: a clinicopathological analysis of 74 cases. Histopathology.

[10] Fergusson IL (1972). Haemangiomata of skeletal muscle. Br J Surg.

[11] To EW, Tsang Wm, Pang PC, Ahuja A (2001). Cavernous hemangioma of the temporalis muscle: report of a case. J Oral Maxillofac Surg.

[12] Bui-Mansfield LT, Myers CP, Fellows D, Mesaros G (2002). Bilateral temporal fossa hemangiomas. AJR Am J Roentgenol.

[13] Odabasi AO, Metin KK, Mutlu C, Başak S, Erpek G (1999). Intramuscular hemangioma of the masseter muscle. Eur Arch Otorhinolaryngol.

[14] Clemis JD, Briggs DR, Changus GW (1975). Intramuscular hemangioma in the head and neck. Can J Otolaryngol.

[15] Heckl S, Aschoff A, Kunze S (2002). Cavernous hemangioma of the temporal muscle. Neurosurg Rev.

[16] Buetow PC, Kransdorf MJ, Moser RP Jr, Jelinek JS, Berrey BH (1990). Radiologic appearance of intramuscular hemangioma with emphasis on MR imaging. AJR Am J Roentgenol.

[17] Lopez-Cedrun JL, Urtasun Fernandez J, Melendez Baltanas J, Lopez Garcia JA (1996). Hemangioma of the temporalis muscle: a case report and review of the literature. J Oral Maxillofac Surg.

[18] Sherman JA, Davies HT (2001). Intramuscular hemangioma of the temporalis muscle. J Oral Maxillofac Surg.

[19] Gadhia K, Bunyan R, Chan CH (2011). Multiple radio-opacities in an OPG: a case report of cavernous haemangioma of temporalis muscle with multiple phleboliths. Dent Update.

[20] Sharma RR, Netalkar AS, Pawar SJ, Musa MM, Lad SD (2001). Congenital and late-onset primary haemangiomas of the temporalis muscle: case report. Br J Oral Maxillofac Surg.

[21] Joehl RJ, Miller SH, Davis TS, Graham WP 3rd (1979). Hemangioma of the temporalis muscle: a case report and review of the literature. Ann Plast Surg.

[22] Sharma BS, Chari PS, Joshi K, Rajvanshi A (1991). Hemangioma of the temporalis muscle. Ann Otol Rhinol Laryngol.

[23] Murakami M, Nonaka N, Hirata Y, Sonoda H, Ushio Y (1991). Hemangioma of the temporalis muscle: case report and review of the literature. Surg Neurol.

[24] Hughes C, Hutchison I (1993). Temporalis haemangioma presenting as temporomandibular joint pain dysfunction syndrome. Br J Oral Maxillofac Surg.

[25] Cappabianca P, Cirillo S, de Divitiis E, de Caro MB, Spaziante R, Zona G (1996). Hemangioma of the temporal muscle. Head Neck.

[26] Tada M, Sawamura Y, Abe H, Itoh F, Saito H, Nagashima K (1996). Venous hemangioma of the temporalis muscle. Neurol Med Chir (Tokyo).

[27] Couloigner V, Levy J, Laxenaire A, Roucayrol AM, Lerondeau JC, Scheffer P (1996). Venous hemangioma of the temporal muscle. Review of the literature: apropos of a case (Article in French). Rev Stomatol Chir Maxillofac.

[28] Shpitzer T, Noyek AM, Witterick I (1997). Noncutaneous cavernous hemangiomas of the head and neck. Am J Otolaryngol.

[29] Itosaka H, Tada M, Sawamura Y, Abe H, Saito H (1997). Vanishing tumor of the temporalis muscle: repeated hemorrhage in an intramuscular venous hemangioma. Am J Neuroradiol.

[30] Benateau H, Labbé D, Kaluzinski E, Théron J, Mandard JC, Compère JF (1997). Intramuscular capillary-venous angioma extending into the infratemporal fossa. Apropos of a case (Article in French). Rev Stomatol Chir Maxillofac.

[31] Top H, Barcin E (2004). Posttraumatic intramuscular hemangioma of the left temporal muscle. Eur J Plast Surg.

[32] Sakr A, Kasem M, Khalil H, Khan F, Nasta N (2005). Cavernous haemangioma of the temporalis muscle: a case report and review of the literature. Eur. J. Radiol.

[33] Calişaneller T, Ozdemir O, Yildirim E, Kiyici H, Altinörs N (2007). Cavernous hemangioma of temporalis muscle: report of a case and review of the literature. Turk Neurosurg.

[34] Bucci T, De Giulio F, Romano A, Insabato L, Califano L (2008). Cavernous haemangioma of the temporalis muscle: case report and review of the literature. Acta Otorhinolaryngol Ital.

[35] Kim JM (2009). Intramuscular hemangioma of temporal muscle. Korean J Otorhinolaryngol-Head Neck Surg.

[36] Eryilmaz MA, Varsak YK, Gül Z, Uğur A (2014). Intramuscular cavernous hemangioma of the temporalis muscle. J Craniofac Surg.

[37] Kim JH, Lew BL, Sim WY (2014). Intramuscular vascular malformation of the temporalis muscle: a case report and review of the literature. Ann Dermatol.

[38] Turel MK, Kiehl TR, Gentili F (2016). Extracranial temporal cavernous hemangioma: differential diagnosis, and a review of literature. Neurol India.

[39] Cui B, Wang DH, Wang GJ, Cheng P, Zhang F, Duan XB, Zhao ZF (2017). Cavernous hemangiomas of the temporalis muscle with prominent formation of phleboliths: case report and review of the literature. Medicine (Baltimore).

[40] Arora N, Bhargava EK, Nambillath AK, Meher R (2017). Intramuscular capillary haemangioma of the temporalis muscle: a rare case with a review of the literature. J Clin Diagn Res.

[41] Kishimoto T, Sukegawa S, Katase N (2019). Endoscope-assisted resection of intramuscular cavernous hemangioma within the temporal muscle. J Craniofac Surg.

[42] S. Jbali, S. Kedous, H. Farah (2018). Temporalis muscle’s cavernous hemangioma: a new case report and review of the literature. J Tun Orl.

[43] Motazedian G, Khojasteh A, Motazedian N, Anbardar MH (2020). Cavernous hemangioma of temporalis muscle: a case report. World J Plast Surg.

[44] Watanabe H, Osano H, Naitou H, Mori Y (2020). A case of cavernous hemangioma of the temporalis muscle. J Oral Maxillofac Surg Med Pathol.

